# Evaluating the impact of microalgae powder on physicochemical and functional properties of hard candies

**DOI:** 10.1007/s10068-026-02174-0

**Published:** 2026-06-03

**Authors:** Shafia Maryam, Berkay Berk, Hilmi Eriklioglu, Baris Ege Gulenc, Kosain Kousar, Mecit Halil Oztop, Muhammad Qasim Hayat, Sarper Dogdu, Mehmet Ali Marangoz, Hussnain Ahmed Janjua

**Affiliations:** 1 Department of Microbiology and Biotechnology, Atta-ur-Rahman School of Applied Biosciences, National University of Science and Technology (NUST), Islamabad, 44000 Pakistan; 2 Food Engineering Department, Middle East Technical University, 06800 Ankara, Turkey; 3 Department of Plant Biotechnology, Atta-ur-Rahman School of Applied Biosciences, National University of Science and Technology, Islamabad, 44000 Pakistan; 4 Durukan Sekerleme, 06935 Ankara, Turkey; 5 Department of Food Engineering, Izmir Institute of Technology, 35433 Izmir, Turkey

**Keywords:** Algae biomass, Hard candy, Functional food, Antioxidant, Scanning electron microscopy, Nuclear Magnetic Resonance

## Abstract

This study investigated the feasibility of incorporating microalgae powders into model hard candies and evaluated their effects on physicochemical, structural, and functional properties. Microalgae species including *Chlorella vulgaris*, *Spirulina platensis*, *Dictyosphaerium* sp., and *Pectinodesmus* sp. were incorporated into candy formulations. Scanning electron microscopy revealed a semi-smooth candy surface with algal particles embedded within the amorphous sugar matrix. Time-domain nuclear magnetic resonance and hardness analysis indicated reduced crystallization in algae-enriched candies. Fourier-transform infrared spectroscopy identified characteristic polysaccharide bands along with amide and protein-associated functional groups derived from algal biomass. Biochemical analyses confirmed the presence of antioxidant activity, phenolic compounds, and flavonoids. Shelf-life evaluation demonstrated the retention of structural and textural stability over three months of storage. Microalgae incorporation influenced candy color, crystalline structure, moisture content, and nutritional composition while enhancing antioxidant functionality. This study provides a basis for the development of microalgae-fortified functional confectionery products.

**Graphical abstract:**

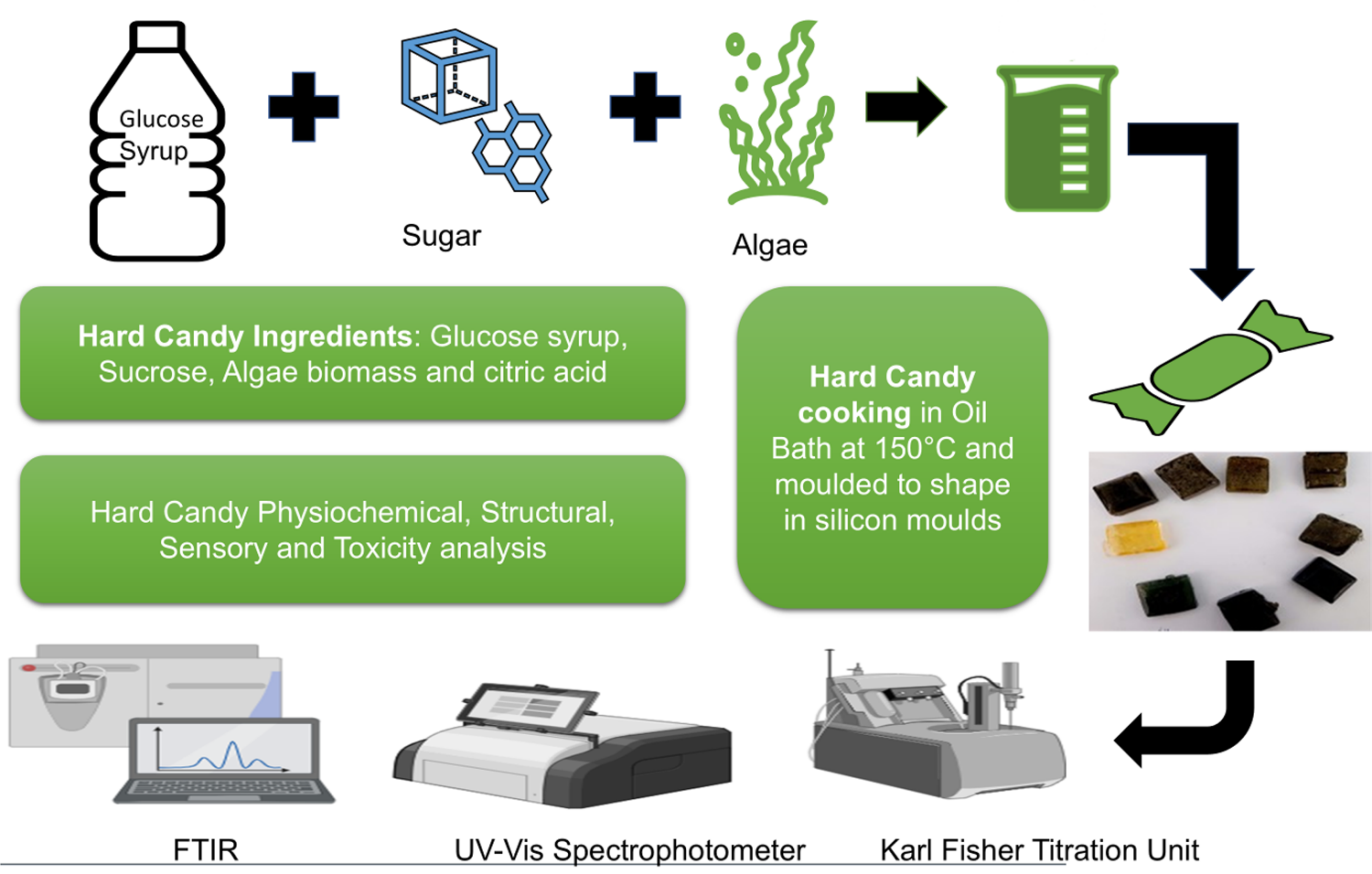

**Supplementary Information:**

The online version contains supplementary material available at 10.1007/s10068-026-02174-0.

## Introduction

The increasing demand for functional foods has positioned microalgae as a promising ingredient for food fortification. Globally, algae contribute approximately 30% of aquaculture production and are recognized as sustainable sources of proteins, lipids, vitamins, and antioxidants for human nutrition (Araújo et al., 2021). Microalgal biomass is particularly valued for its high content of bioactive compounds, including pigments such as mycosporine, β-carotene, and astaxanthin, which are known to confer significant in vitro antioxidant activity (Dam et al., 1965). Incorporation of these compounds into food matrices represents an effective strategy for the development of functional foods.

To date, algal biomass has been successfully incorporated into a wide range of products, including dairy items, baked foods, beverages, and confectionery products such as soft candies (Boukid and Castellari, 2021). *Spirulina platensis* combined with pomegranate peels has been used to enhance the nutritional values of hard candies (Cheliabiieva and Buialska, 2022), while *Spirulina platensis* extracts have improved the physicochemical and antioxidant properties of ice cream (Durmaz et al., 2020). Moreover, the inclusion of microalgae into food products is shown to enhance their functional profile by imparting anti-inflammatory, cardiovascular, and immunomodulatory effects (Sidari and Tofalo, 2019).

Despite these advances, several challenges hinder the commercialization of algae-based functional foods, including structural instability, reduced bioavailability of bioactive compounds, and sensory trade-offs. Thermal processing can lead to the degradation of sensitive bioactive components, thereby limiting their bioavailability (Ozel et al., 2024). Furthermore, data regarding the absorption and utilization of microalgal nutrients in the human diet remain limited. Comprehensive studies addressing these knowledge gaps are therefore essential to support the successful development and commercialization of algae-based functional food products.

Hard candies, conventionally prepared by heating sugar syrups to approximately 160 ℃, represent an innovative and suitable vehicle for microalgae fortification. The amorphous structure and extended shelf life make them an ideal carrier for relatively heat-stable, algae-derived nutrients (Ozel et al., 2024).

While previous studies have primarily focused on the incorporation of *Spirulina* into confectionery products, a systematic comparison of diverse microalgae species including underutilized strains from the genera *Dictyosphaerium* and *Pectinodesmus*. The data on their comprehensive effects on hard candy properties is lacking (Boukid and Castellari, 2021).

This study aims to fill this gap by providing a comparative evaluation of how microalgae powders from different species influence the physicochemical, structural, nutritional, antioxidant, and preliminary safety profiles of model hard candies. In particular, the relationship between algal composition (e.g., protein content, lipid content, and particle size) and critical candy properties such as texture, crystallinity, and thermal behavior were examined. The application of nuclear magnetic resonance spectroscopy in this study provides molecular-level insight into algal fortified hard candy systems through a non-destructive analytical approach, which has only been scarcely explored in confectionery matrices. Collectively, this study offers new insights to support the rational design of microalgae-fortified functional confectionery products.

## Materials and methods

### Chemicals

Sucrose (brand: Balküpü) and glucose syrup (brand: Glukoz Şurubu, Cargill) were purchased in Türkiye. All other chemicals used in the study were obtained from the Sigma Chemical Co. (St Louis, MO, USA).

### Sample preparation

Microalgae samples (*Dictyosphaerium* sp. DHM1, *Dictyosphaerium* sp. DHM2, *Pectinodesmus* sp. PHM3) were collected, identified, and reported (Khalid et al., 2017). *Dictyosphaerium* sp. HSYM was isolated from wastewater collected by Fauji Fertilizers. The strain *Dictyosphaerium* sp. HS was collected by Institute of Environmental Engineering and Science, NUST, Islamabad.

All strains were cultivated on Bold’s Basal Medium (BBM). Microalgal inoculum was grown in flasks under illumination from white, fluorescent lamps. A continuous 24-h light cycle at 100 μmol photons m^−2^ s^−1^ was provided. Aeration was supplied using air pumps and the temperature was maintained between 20 and 30 ℃. Microalgal biomass was harvested after 30 days and centrifuged at 3000 rpm for 5 min. Centrifugation was repeated 4 times to remove residual BBM media.

The biomass was dried in an oven at 40 ℃ for 24 h. Dried biomass was crushed and sieved through a series of cascaded stainless-steel sieves (Glennamer Model: N/A-237) following mechanical agitation. The mass retained on each sieve was measured to calculate the percentage of particles within specific size ranges (5–35 µm) (Prasedya et al., 2021).

Commercial *Spirulina platensis* and *Chlorella vulgaris* powders were purchased from the brand Nuturiga. An industrial grade *Spirulina platensis* was obtained from Kayseri Seker, Türkiye that is mentioned as *Spirulina platensis* Kayseri.

### Candy preparation

Hard candies are amorphous, glassy solids traditionally prepared by boiling sugar syrup at 150–160 ℃ to remove water and form a supersaturated solution (Ozel et al., 2024). In this study, candies were prepared using glucose syrup (31.25 g) and sucrose (25 g), resulting in a total mass of 56.25 g. The mixture was heated to 140 ℃. Subsequently, 0.5 g of algae powder and 0.2 mg of citric acid were incorporated and mixed thoroughly.

The mixture was poured into molds, allowed to cool, and stored in airtight aluminium bags. Candies were kept under dark, dry conditions at room temperature (25 ℃). Prior to analysis, candies were ground and sieved into particle size fractions 250–500 μm (for texture, color, hardness, fracturability, and brittleness analysis), 100–250 μm (for FTIR, ºBrix, and compositional analyses), and < 100 μm (for NMR and MTT assays) using stainless steel sieves (Endecotts Ltd.).

## Physicochemical analysis

### Color evaluation

Color of the algae biomass and hard candies was measured using bench top spectrophotometer (Data color 110™, Lawrenceville, NJ, USA). The instrument was calibrated using a standard white plate. Color values were expressed using the CIELAB system: L* (lightness), a* (redness/greenness), and b* (yellowness/blueness). Measurements were conducted in triplicate at three different locations on each sample at room temperature and mean values were recorded. Color measurements were performed on the first day of the storage (Kamiloglu et al., 2015).

### Texture analysis

Candy hardness was measured using a Shore A hardness tester. The durometer was mounted vertically, with a metal indenter applying a force of 75 mN. Brittleness and fracturability were evaluated using a Universal testing machine with 0.5 N load cell and a test speed of 1 mm s^−1^. All measurements were performed in triplicates on the first day of storage (Mohamed and Aggag, 2003).

### Glass transition temperature (Tg)

Glass transition temperature was determined using a DSC 4000 instrument (Perkin Elmer, MA, USA). Nitrogen gas flow was maintained at 19.8 mL min^−1^. Approximately 10 mg of samples were sealed in hermetic aluminium pans are heated from−2 to 100 ℃ at a rate of 10 ℃ min^−1^. T_g_ values were determined using Pyris Manager software. Measurements were performed in triplicate on the first day of the storage (Tau and Gunasekaran, 2016).

### ºBRIX values

Soluble solid content was measured using a refractometer (SCM-1000, HM DIGITAL, Seoul, Korea) and results are expressed in ^o^Brix. Measurements were recorded in triplicate on the first day of storage (Kuzu et al., 2025).

### Organoleptic analysis

A total of 20 volunteers participated in a preliminary acceptance test for organoleptic evaluation (Lesschaeve and Noble, 2022). Evaluations were conducted in two sessions per week on alternate days. Nine candy samples were randomly presented in white plastic dishes during each session. Sessions were conducted in individual booths under fluorescent light.

Samples were evaluated for overall acceptance, texture, color, aroma, hardness, shine, green hue, transparency, and stickiness using a 9-point hedonic scale from 1 (extremely dislike) to 9 (extremely like). Panellists were blindfolded during texture, stickiness, and aroma evaluation. Samples used in this test were not intended for consumption and contained components unsuitable for skin or mucosal exposure; therefore, all panelists wore single-use nitrile gloves.

Principal Component Analysis (PCA) was applied to reduce dimensionality and simplify multivariate data interpretation. Nine samples and ten variables were included. Components with eigenvalues greater than 1 were retained and the first two Principal Components (PC) were used. PCA was performed using a correlation matrix and analyses were conducted using Minitab 19 software.

## Structural analysis

### Scanning electron microscopy analysis

Hard candy samples were analysed using a field emission scanning electron microscope (FESEM) to examine surface morphology and cross-sections. Samples were analysed at an accelerating voltage of 10 kV. Upper surface, lower surface, and cross-sectional regions were examined (Naidoo et al., 2020).

### Fourier transform infrared (FTIR) spectroscopy

Samples were analysed using an IR Affinity-1 spectrometer equipped with an attenuated total reflectance (ATR) attachment (Shimadzu Corporation, Kyoto, Japan). Spectra were recorded over a range of 4000–600 cm^−1^ for 10 scans at a resolution of 4 cm^−1^. Spectral data were processed using Origin Pro 9.0 software (Namli et al., 2021).

### X-ray diffraction (XRD)

Crystallinity was analysed using a Rigaku Ultima-IV X-ray diffractometer (The Woodlands, USA). Samples were prepared in 1—mm thick specimens. Measurements were conducted using a scan width of 0.02°, a 2θ range of 3°–80°, and a scan rate of 2° min^−1^ (Qiao et al., 2017).

### Time domain nuclear magnetic Resonance (TD-NMR)

TD-NMR experiments were conducted using a low-resolution 0.5 T (20.34 MHz) (Spin Track, Resonance Systems GmbH, Germany) equipped with a 10—mm RF coil. Candy samples were crushed and transferred into NMR tubes for analysis (Kuzu et al., 2025).

### Longitudinal relaxation time (T_1_) measurements

T_1_ measurements were performed using saturation recovery sequence with a relaxation period of 2 s, delay times of 1000–1500 ms, 16 data points, and four scans. Mono and multi-exponential fitting of relaxation curves was performed using Relax8 software (Resonance Systems GmBH) (Kuzu et al., 2025).

### Solid echo Measurements for relative crystallinity

Solid echo measurements were conducted using the same TD-NMR system. Second-moment values were obtained using a solid echo sequence with a repetition delay of 10 s and 32 scans. Data were processed using the “Solid Lab” module in Relax8 software. Measurements were conducted in triplicate (Kuzu et al., 2025).

## Antioxidant analysis

### Radical scavenging activity

Antioxidant activity was determined using the DPPH assay (Hangun-Balkir and McKenney, 2012), with Trolox as the standard. Distilled water served as the control for algal biomass and control candy without algae was used for candy samples. Candies were crushed and sieved through a 0.5mm mesh. Absorbance was measured at 517nm using a UV/VIS Spectrophotometer (Optizen Pop Nano Bio, Mecasys Co. Ltd, Korea). Results were expressed as Trolox equivalents and percentage DPPH radical scavenging using the following equation:

DPPH scavenging activity (%) = (A control-A sample) / A control × 100.

### Total reducing power

Reducing power was measured by ferricyanide/Prussian blue method and results were expressed as Trolox equivalent. Measurements were performed in triplicates (Işıl Berker et al., 2010).

### Total phenolic content

Total phenolic content was determined using the Singleton and Rossi method (Singleton and Rossi, 1965). Absorbance was measured at 765 nm and results were expressed as gallic acid equivalents (mg/g).

### Total flavonoid content

Total flavonoid content was determined using the method described by Mahboubi (Mahboubi et al., 2013), with catechin as the standard. Absorbance was measured at 450nm and results were expressed as mg/g.

### Nutritional composition

The nutritional composition of algae biomass and algae-enriched hard candies was evaluated to assess the transfer of nutrients from algae to the candy matrix. Protein, carbohydrate, lipid, mineral, and moisture contents were quantified and expressed as percentages.

### Moisture content determination

Moisture content was determined by Karl Fischer titration (TitraLab KF1000 Series, HACH, UK) at 25 ℃. Measurement were conducted on the second day of storage using a two-component system in triplicates (Kuzu et al., 2025).

### Estimated carbohydrate content

Carbohydrate content was estimated using the Loewus anthrone method by measuring absorbance at 620 nm, with glucose as the standard (A and E labs, Model AE-S902D Serial No. AT1604003) (Loewus, 1952). Analysis were performed in triplicates.

### Protein content

Protein contents was measured using Lowry method with bovine serum albumin as the standard (Lowry et al., 1951). Analysis was performed in triplicates.

### Mineral content

Ash content was determined using a gravimetric method according to AOAC Method 08–01. Samples were incinerated in a muffle furnace at 550℃ (Carbolite CWF 1200, Serial No: 20-901,274). Algal biomass was ashed for 4 h, while candy samples were ashed for 25 min. Samples were cooled in a desiccator before weighing. Analyses were conducted in triplicate (Thiex et al., 2012).

### Lipid content

The lipid content was determined using the Bligh and Dyer method (Bligh and Dyer, 1959). Lipids were extracted using a chloroform: methanol: water mixture (5:10:4, v/v/v). Samples were incubated overnight at 37 °C, centrifuged at 6000 rpm for 10–15 min, and lipid fractions were collected, dried, and weighed. Analyses were performed in triplicates.

### Preliminary cytocompatibility analysis

Preliminary cytocompatibility of algae-enriched candies was evaluated using the MTT assay on HEK-293 cells. Candy samples were diluted in PBS and tested at concentrations of 100, 70, and 30%. Cells were cultured in DMEM and incubated at 37 ℃ under 5% CO_2_ and 95% air. After exposure, cells were incubated with MTT solution (250 μg m/L) for 3 h. Formazan crystals were dissolved in DMSO, and absorbance was measured at 580 nm (Jalilian et al., 2021).

### Shelf-life analysis

Candies were packaged in metallized polyester films and stored in a dark, dry environment at 22℃ with 45–60% relative humidity for three months. Hardness, color, moisture content, and antioxidant activity were analyzed periodically using the DPPH assay with Trolox as the standard(Hangun-Balkir and McKenney, 2012).

### Experimental design and replication

Three independent candy batches were produced on separate days as biological replicates for the following analyses: color, texture, T_g_, °Brix, organoleptic evaluation, TD-NMR, antioxidant activity/content, total phenolic content, total flavonoid content, proximate nutritional composition, and preliminary cytocompatibility assessment. All measurements were performed in technical triplicate for each batch. SEM, FTIR, XRD, and shelf-life analyses were conducted using a representative batch under controlled storage conditions.

### Statistical analysis

All results were expressed as mean ± standard deviation (SD). Data were analyzed using RStudio (2025.05.1 Build 513) and Minitab 19. One-way ANOVA, Tukey’s post-hoc test, and PCA were applied. Exact F and p values are provided in Supplementary Tables S1 and S2.

## Results and discussion

### Physicochemical analysis

The incorporation of microalgae powders significantly altered the physicochemical properties of the model hard candies. These effects were attributable to the compositional and physical characteristics of each algal biomass.

One of the most pronounced changes was the dark-green coloration imparted to the candies, as reflected by the L*, a*, and b* color parameters (Table 1). These changes were governed by the pigment composition of the respective algal powders. For example, *Spirulina platensis* Kayseri (Spk) powder produced candies with the lowest L* (darkest) and the most negative a* (most green) values (Table 2). This result confirmed efficient transfer of algal pigments into the candy matrix and demonstrated that the intrinsic color of the algal biomass primarily determined the final visual appearance of the candies.

**Table 1:** Physicochemical (Color L*, a*, and b*), textural (hardness, brittleness, and fracturability), Glass Transition Temperature (T_g_), compositional (carbohydrates, protein, minerals, moisture, lipids, and others), and cytocompatibility properties (MTT 100%, 70%, 30%) of the candy samples

Parameters	C	Spk	Ch	Sp	DHM1	DHM2	PHM3	DHSYM		DHS	p-value
Color L*	42.08 ± 0.05^A^	33.9 ± 0.0^B^	24.78 ± 0.0^H^	24.08 ± 0.01^I^	27.65 ± 0.01^F^	29.72 ± 0.01^D^	30.24 ± 0.01^C^		27.88 ± 0.005^E^	27.43 ± 0.01^G^	< 0.0001
Color a*	− 0.77 ± 0.02^C^	− 4.16 ± 0.02^G^	− 0.34 ± 0.34^A^	− 0.64 ± 0.02^B^	− 0.86 ± 0.02^D^	− 1.06 ± 0.01^E^	− 1.6 ± 0.01^F^		− 6.3 ± 0.01^B^	− 0.82 ± 0.02^C^	< 0.0001
Color b*	18.2 ± 0.05^A^	5.26 ± 0.01^C^	1.64 ± 0.02^H^	1.22 ± 0.04^I^	2.65 ± 0.01^F^	6.28 ± 0.02^B^	4.69 ± 0.02^D^		2.79 ± 0.01^E^	1.71 ± 0.01^G^	< 0.0001
Hardness (kgF)	15.5 ± 0.5^AB^	15.67 ± 0.38^AB^	14.33 ± 0.63^B^	15.25 ± 0.66^AB^	15.75 ± 0.43^A^	15.42 ± 0.52^AB^	15.75 ± 0.25^A^		15.75 ± 0.5^A^	15.92 ± 0.38^A^	0.0307
Brittleness (N/mm^2^)	3.9 ± 0.2^D^	5.68 ± 0.04^B^	4.15 ± 0.1^D^	4.3 ± 0.2^D^	4.23 ± 0.15^D^	2.3 ± 0.2^F^	2.97 ± 0.15^E^		6.3 ± 0.2^A^	5.2 ± 0.1^C^	< 0.0001
Fracturability (N/mm^2^)	4.9 ± 0.1^EF^	11.1 ± 0.1^A^	5.4 ± 0.4^E^	5.5 ± 0.5^E^	4.2 ± 0.1^F^	7.4 ± 0.3^C^	8.3 ± 0.1^B^		6.3 ± 0.1^D^	8.9 ± 0.2^B^	< 0.0001
Tg (℃)	39.42 ± 1^A^	35.63 ± 2^BC^	30.86 ± 1^D^	38.29 ± 1^AB^	33.5 ± 1^CD^	34.22 ± 1^C^	33.93 ± 1^CD^		35.86 ± 1^BC^	35.69 ± 1^BC^	< 0.0001
ºBrix	91.36 ± 2.34^B^	91.89 ± 0.02^AB^	95.2 ± 0.52^AB^	94.95 ± 0.3^AB^	95.88 ± 1.84^A^	91.74 ± 1.71^AB^	91.41 ± 2.52^B^		90.79 ± 1.4^B^	91.52 ± 1.04^AB^	0.0024
T_1_ (ms)	175.75 ± 6.8^C^	185.2 ± 0.26^C^	144.5 ± 8.93^D^	176.5 ± 8.8^C^	229.45 ± 5^A^	211.66 ± 2.1^B^	212.08 ± 5.8^B^		234.78 ± 4^A^	224.34 ± 6.06^AB^	< 0.0001
Second Moment (M^2^ 10^8^ Hz^2^)	11.04 ± 0.23^A^	10.52 ± 0.17^A^	10.54 ± 0.18^A^	11.27 ± 0.31^A^	10.56 ± 0.1^A^	10.53 ± 0.1^A^	10.55 ± 0.2^A^		10.85 ± 0.2^A^	10.98 ± 0.3^A^	0.0084
Crystallinity (%)	45.3 ± 2.08^A^	40.8 ± 1.8^A^	40.83 ± 1.6^A^	44.37 ± 2.4^A^	41 ± 0.6^A^	40.73 ± 0.9^A^	37.97 ± 6.7^A^		43.6 ± 1.73^A^	44.77 ± 2.34^A^	0.0658
Carbohydrates (%)	95.34 ± 0.26^A^	92.43 ± 0.15^CDE^	93.23 ± 0.15^B^	92.4 ± 0.27^CDE^	93.23 ± 0.23^BC^	92.33 ± 0.21^DE^	93.1 ± 0.61^BCD^		92.17 ± 0.25^E^	93.13 ± 0.35^BCD^	< 0.0001
Protein (%)	0 ± 0^F^	0.52 ± 0.01^CD^	0.7 ± 0.0^A^	0.56 ± 0.0^B^	0.54 ± 0.0^BC^	0.51 ± 0.01^D^	0.51 ± 0.0^D^		0.47 ± 0.01^E^	0.51 ± 0.0^D^	< 0.0001
Minerals (%)	0.89 ± 0.02^C^	0.49 ± 0.03^E^	0.57 ± 0.061^D^	0.65 ± 0.0^D^	0.65 ± 0.0^D^	0.65 ± 0.0^D^	0.62 ± 0.024^D^		2.83 ± 0.02^A^	0.95 ± 0.0^B^	< 0.0001
Moisture (%)	3.63 ± 0.02^D^	4.23 ± 0.15^BC^	5.2 ± 0.0^A^	4.31 ± 0.01^BC^	2.99 ± 0.11^F^	3.03 ± 0.02^DE^	3.52 ± 0.07^DE^		3.42 ± 0.03^E^	4.06 ± 0.06^C^	< 0.0001
Lipids and Others (%)	0.13 ± 0.23^E^	2.32 ± 0.33^B^	0.3 ± 0.2^E^	2.1 ± 0.27^BC^	2.59 ± 0.15^AB^	3.48 ± 0.19^BC^	2.25 ± 0.67^BC^		1.12 ± 0.27^D^	1.35 ± 0.39^CD^	< 0.0001
MTT 100% (%)	89.03 ± 0.83^A^	83.37 ± 0.42^BC^	84.73 ± 0.64^B^	80.27 ± 1.02^D^	80.03 ± 1^D^	84.17 ± 1^B^	81.3 ± 0.58^CD^		84.47 ± 0.5^B^	80.67 ± 0.6^D^	< 0.0001
MTT 70% (%)	84.73 ± 0.64^AB^	81.13 ± 1^CD^	83.53 ± 0.5^BC^	80.4 ± 0.53^D^	86.23 ± 1.08^A^	84.27 ± 1.1^AB^	80.87 ± 1^D^		80.67 ± 0.6^D^	81 ± 1^D^	< 0.0001
MTT 30% (%)	84.17 ± 0.76^A^	81.5 ± 2.18^AB^	82.4 ± 0.53^AB^	84.3 ± 0.61^A^	80.3 ± 0.6^B^	82.17 ± 2^AB^	83.2 ± 1.06^AB^		83.2 ± 0.7^AB^	83.93 ± 1^AB^	0.0173

Samples are coded as follows: *C* Control, *Spk* *Spirulina platensis* Kayseri, *Ch* *Chlorella vulgaris*, *Sp* *Spirulina platensis*, *DHM*1 *Dictyosphaerium* HM1, *DHM*2 *Dictyosphaerium* HM2, *PHM*3 *Pectinodesmus pectinus* HM3, *DHSYM* *Dictyosphaerium* HSYM, *DHS* *Dictyosphaerium* HS

^1^Values are presented as mean ± standard deviation (n = 3). Different superscript letters within a row indicate significant differences (Tukey HSD test)

^2^Significance levels were defined as p < 0.05 (significant), p < 0.01 (highly significant), and p < 0.001 (extremely significant)

**Table 2:** Color, particle size, and proximate composition (carbohydrates, protein, minerals, moisture, and lipids) of algae biomass samples

Parameters	Spk	Ch	Sp	DHM1	DHM2	PHM3	DHSYM	DHS
Color L*	44.53 ± 0.25^B^	42.6 ± 0.1^C^	44.63 ± 0.05^B^	41.6 ± 0.1^D^	40.83 ± 0.47^E^	42.4 ± 0.1^C^	45.43 ± 0.25^A^	42.43 ± 0.1^C^
Color a*	–15.27 ± 0.12^B^	−12.1 ± 0.1^A^	–15.47 ± 0.12^B^	−12.4 ± 0.1^A^	−12.1 ± 0.1^A^	–12.23 ± 0.17^A^	−12.2 ± 0.17^A^	−12.2 ± 0.1^A^
Color b*	–11.37 ± 0.12^B^	28.5 ± 0.24^A^	–11.3 ± 0.08^B^	28.13 ± 0.12^A^	28.27 ± 0.12^A^	28.27 ± 0.12^A^	28 ± 0.22^A^	28.3 ± 0.14^A^
Particle Size (microns)	23.67 ± 3.1^B^	6.7 ± 2.9^C^	22.3 ± 1.7^B^	41.67 ± 2.87^A^	54.33 ± 7.59^A^	46 ± 0.82^A^	44.33 ± 4.6^A^	45.33 ± 3.1^A^
Carbohydrates (%)	20.37 ± 0.01^E^	18.35 ± 0.01^F^	20.3 ± 0.02^E^	32.92 ± 0.01^B^	33.74 ± 0.02^D^	27.98 ± 0.01^D^	32.78 ± 0.01^B^	30.86 ± 0.01^C^
Protein (%)	45.27 ± 0.02^C^	50.93 ± 0.1^A^	47.1 ± 0.03^B^	31.79 ± 0.02^A^	37.87 ± 0.04^D^	34.98 ± 0.01^E^	32.37 ± 0.01^G^	34.11 ± 0.01^F^
Minerals (%)	6.77 ± 0.38^A^	5.6 ± 0.2^B^	6.5 ± 0.3^A^	6.57 ± 0.15^A^	6.67 ± 0.15^A^	5.73 ± 0.35^B^	6.5 ± 0.17^A^	6.53 ± 0.15^A^
Moisture (%)	3.47 ± 0.39^B^	3.0 ± 0.12^B^	3.33 ± 0.17^B^	6.97 ± 0.17^A^	6.87 ± 0.66^A^	6.47 ± 0.4^A^	7.03 ± 0.17^A^	7.5 ± 0.36^A^
Lipids (%)	9.87 ± 0.21^F^	11.53 ± 0.35^D^	9.73 ± 0.21^F^	14.6 ± 0.61^B^	11.97 ± 0.38^D^	21.57 ± 0.38^A^	12.57 ± 0.57^C^	10.83 ± 0.32^E^

Samples are coded as follows: *Spk* *Spirulina platensis* Kayseri, *Ch* *Chlorella vulgaris*, *Sp* *Spirulina platensis*, *DHM*1 *Dictyosphaerium* HM1, *DHM*2 *Dictyosphaerium* HM2, *PHM*3 *Pectinodesmus pectinus* HM3, *DHSYM* *Dictyosphaerium* HSYM, *DHS* *Dictyosphaerium* HS

^1^Values are presented as mean ± Standard deviation (n = 3). Different superscript letters within a row indicate significant differences (*p* < 0.001; Tukey HSD test)

Textural properties were also markedly affected by algal incorporation. A substantial reduction in hardness and glass transition temperature (Tg) was observed in *Chlorella*-enriched candies (Table 1). This behavior was associated with the compositional features of *Chlorella* powder, which exhibited the highest protein content (50.93%) and the smallest particle size (6.7 µm) among the tested samples (Table 2). The presence of low-molecular weight proteins disrupted sucrose-sucrose hydrogen bonding, increased free volume, and enhanced molecular mobility, which softened the candy structure and reduced Tg (Ladjal-Ettoumi et al., 2024).

In contrast, *Spirulina* and *Dictyosphaerium* powders contained lower protein contents and larger particle sizes therefore behaved as inert fillers. Their incorporation caused minimal disruption to the amorphous sucrose network. Hardness and Tg values of these candies remained comparable to those of the control.

This interpretation was further supported by the brittleness and fracturability profiles (Table 1). The highest brittleness observed in *Dictyosphaerium* HSYM (DHSYM) candies was associated with elevated protein and mineral contents, which created additional stress concentration sites within the matrix. Conversely, the lower brittleness observed in *Dictyosphaerium* HM2 (DHM2) and *Pectinodesmus pectinus* HM3 (PHM3) was associated with their higher lipid content (Table 2), which reduced internal friction within the amorphous sugar structure.

Thermal behavior trends were consistent with Tg and °Brix measurements. Control, *Spirulina*, and *Dictyosphaerium* (HS and HSYM) candies exhibited Tg values above 34℃ (Table 1), which reflected enhanced structural stability of the amorphous matrix (Reinheimer et al., 2010). Variations in °Brix, particularly the elevated values observed in *Chlorella*, *Spirulina* and *Dictyosphaerium* DHM1 candies, were attributed to the dissolution of low-molecular-weight algal constituents such as sugars, amino acids, and inorganic ions. These components increased ºBrix relative to the control formulation.

SEM images corroborated these physicochemical findings. The candy surface exhibited a semi-smooth morphology with ridges and depressions (Fig. 2A), while air bubbles were observed on the lower surface (Fig. 2B and 2C) with an average diameter of 409.31 μm. Bubble size varied with cooling duration and longer cooling times produced larger voids. Algal particles were embedded within the candy layers (Fig. 2D) and appeared partially aligned yet randomly dispersed, which confirmed heterogeneous distribution. Particle sizes observed in Fig. 2E–H ranged from 1.9 to 3.6 µm. Together with T_g_ data, SEM analysis confirmed that structural integrity and layered morphology were maintained following microalgae incorporation.

### Organoleptic results

**Fig. 1:**
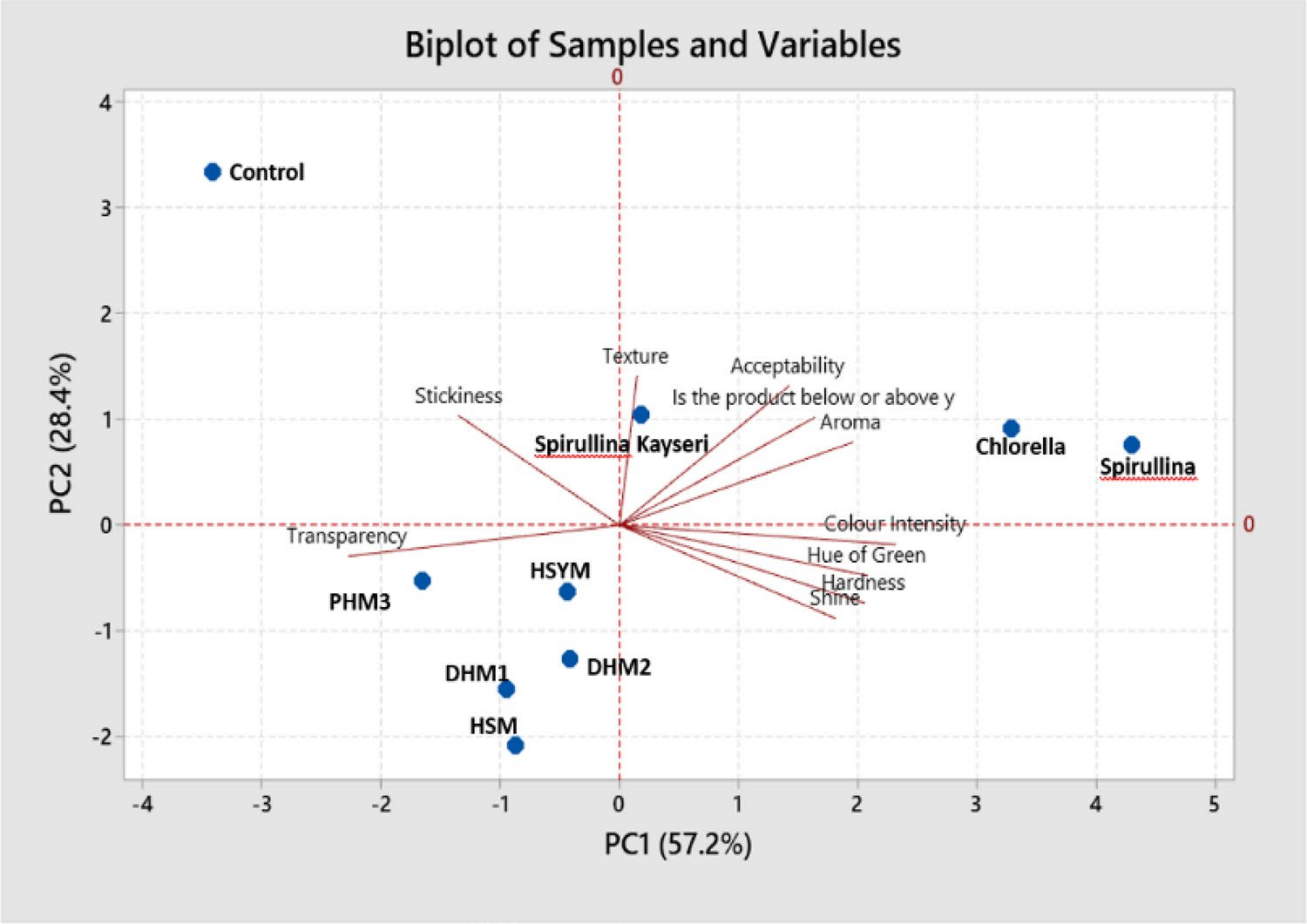
Organoleptic analysis biplot of principal component values for both samples and variables. Samples: Control; Spirullina Kayseri, *Spirulina platensis* Kayseri; Chlorella, *Chlorella vulgaris*; Spirullina, *Spirulina platensis*; DHM1, *Dictyosphaerium* HM1; DHM2, *Dictyosphaerium* HM2; PHM3, *Pectinodesmus pectinus* HM3; HSYM, *Dictyosphaerium* HSYM; HSM, *Dictyosphaerium* HS. PC1 (eigenvalue = 5.72) and PC2 (eigenvalue = 2.84) accounted for 57.2% and 28.4% of the total variance, respectively, reaching a cumulative variance of 85.6%

A consumer-based organoleptic analysis was conducted using a 9-point hedonic scale (Felföldi et al., 2022). The results highlight that *Spirulina* and *Chlorella* enriched candies achieved the highest acceptability, with hedonic totals ranging from 53.4 to 69.8, though differences from the control were not statistically significant (p > 0.05) (Fig. S1A, supplementary file). Principal Component Analysis (PCA) explained 85% of the variance (PC1: 57%, PC2: 28%) presented in Fig. S1B (supplementary file). The biplot in Fig. 1 revealed distinct clustering: *Spirulina* and *Chlorella* grouped together with high positive PC1 scores, associated with favourable attributes; color intensity, hue of green, hardness, and shine. In contrast, PHM3, DHM1, DHM2, HSM, and HSYM are clustered in the negative PC1/PC2 quadrant, while the control sample was positioned separately with high PC2 and negative PC1. Attributes such as acceptability, aroma, and texture were positive on both PCs, whereas stickiness and transparency were less aligned with the main clusters. These results indicate that *Spirulina* and *Chlorella* based candies are strongly linked with desirable visual and textural qualities.

### Structural analysis

The FTIR results demonstrated little variation in the spectra of the functional groups present in different candy samples, examined over a wavelength range of 600–4000 cm^−1^ (Nandiyanto et al., 2019). Peaks at 921–931 cm^−1^ were associated with carbon-related components, while those between 1045 and 1053 cm^−1^ indicate C–O stretching coupled with C–O bending of the C–OH, confirming the presence of carbohydrates. Protein and collagen contents were identified by a peak appearing at 1159–1164 cm^−1^ corresponding to C–O stretching vibrations in hydroxyl groups of serine, threonine, and tyrosine residues, with Amide III confirmed at 1233–1238 cm⁻^1^. Peaks at 1401–1458, 1517–1526, and 1633–1635 cm^−1^ corresponded to Amide II and Amide I bands, while amino-related compounds were detected at 2332–2350 cm⁻^1^. Peaks at 2853–2959 cm^−1^ indicated the presence of fats and those at 3092–3293 cm^−1^ represented alcohols and hydroxyl groups.

**Fig. 2:**
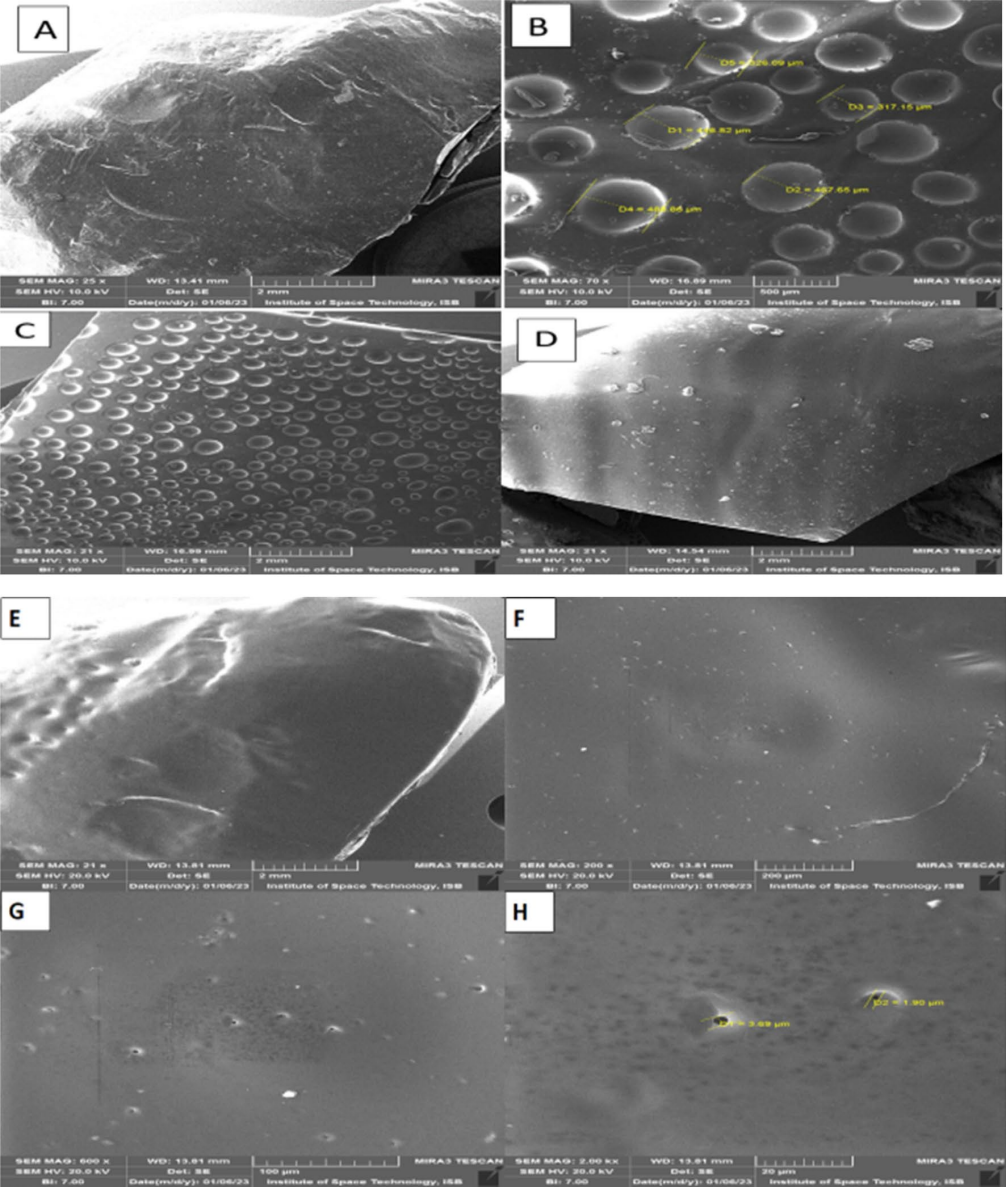

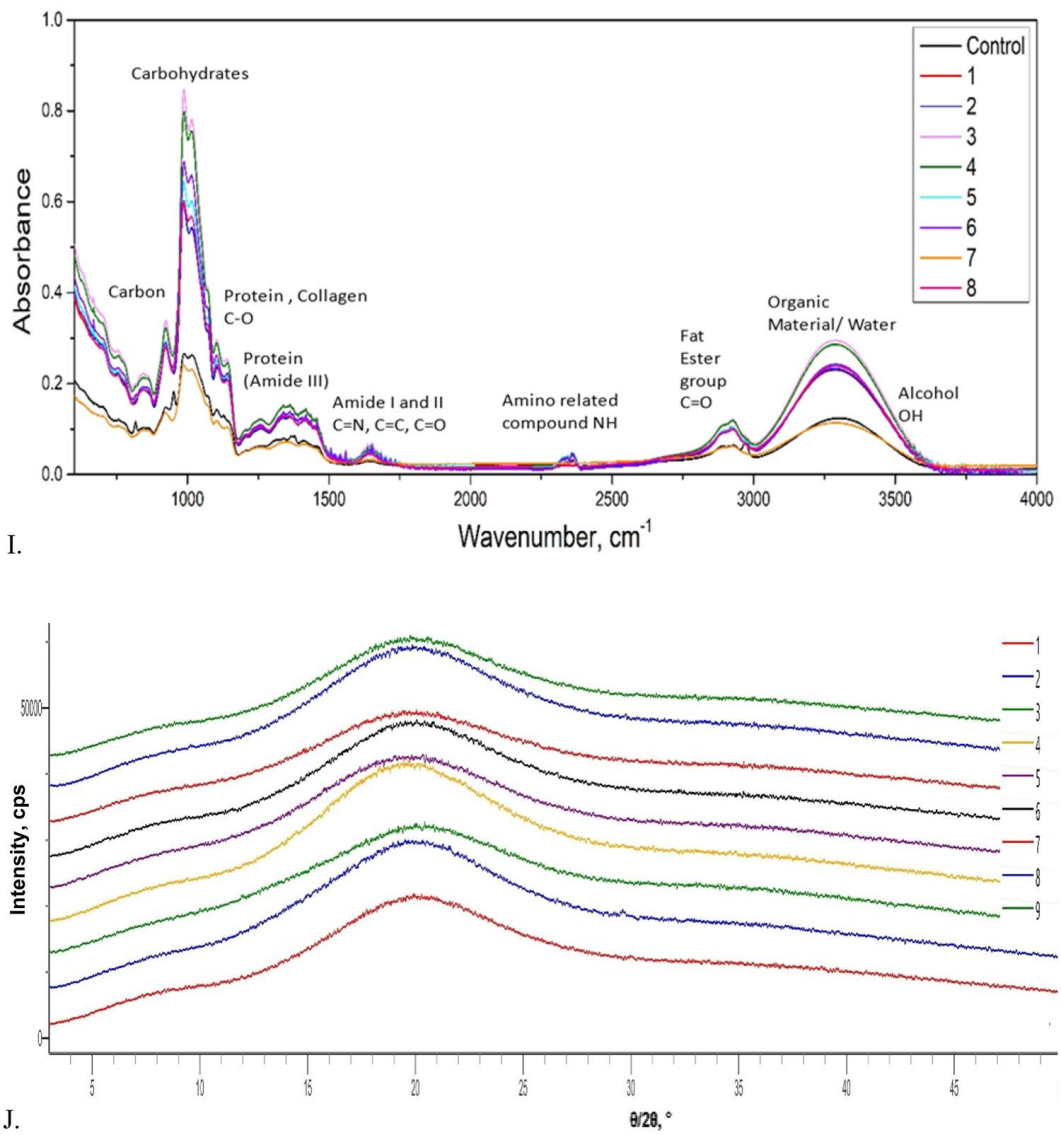
Structural characterization of microalgae-incorporated hard candies: Scanning electron micrographs of microalgae-incorporated hard candies (10 kV acceleration voltage): **(A)** Cross-sectional view reveals characteristic rough ridges (scale bar = 2 mm), **(B)** 70 × magnified back section revealing air bubble distribution (scale bar = 500 μm), **(C)** Back section microstructure with porous morphology (scale bar = 2 mm), **(D)** Front section demonstrating smooth surface topography (scale bar = 2 mm), **(E,F,G,H)** 21–2.0k X magnified cross-sectional view of candy revealing algae particle size of 1.9 and 3.69 µm. All samples were sputter-coated with gold/palladium prior to imaging, **(I)** FTIR spectra revealing functional group vibrations (4000–400 cm^−1^) for Samples: **C**, Control; **1**, *Spirulina platensis* Kayseri; **2**, *Chlorella vulgaris*; **3**, *Spirulina platensis*; **4**, *Dictyosphaerium* HM1; **5**, *Dictyosphaerium* HM2; **6**, *Pectinodesmus pectinus* HM3; **7**, *Dictyosphaerium* HSYM; **8**, *Dictyosphaerium* HS, **(J)** XRD patterns (2θ = 5–50°) of candy samples: **1**, Control; **2**, *Spirulina platensis* Kayseri; **3**, *Chlorella vulgaris*; **4**, *Spirulina platensis*; **5**, *Dictyosphaerium* HM1; **6**, *Dictyosphaerium* HM2; **7**, *Pectinodesmus pectinus* HM3; **8**, *Dictyosphaerium* HSYM; **9**, *Dictyosphaerium* HS

The FTIR spectra (Fig. 2I) showed higher intensities of carbohydrate, protein (Amide I, II, III), fats, water, and alcohol functional groups in all algae-enriched candies (Spectra 1–8) compared to the control. Other than carbohydrates, most peaks appeared small and sharp, indicating the chemical structure within the candies was compact and well-defined. The amino-related NH group was absent in control and in *Dictyosphaerium* sp. HS and HSYM supplemented candy (Spectra 7) displayed a spectrum closest to the control. Complementary XRD analysis (Fig. 2J) confirmed amorphous nature, despite algal biomass (1% w/w) incorporation. The diffractogram exhibited an almost flat profile with no well-defined peaks, confirming the absence of long-range crystalline order and the predominance of an amorphous matrix, with only minor crystalline components present (Lopez‐Rubio et al., 2008).

Longitudinal relaxation time (T_1_) values were used to assess molecular mobility, moisture distribution, and crystallinity (Table 1). The T_1_ data were fitted to a mono-exponential model. *Spirulina* and *Chlorella* enriched candies exhibited lower T_1_ values than *Dictyosphaerium* and *Pectinodesmus* based formulations. These results reflected greater molecular mobility and reduced structural rigidity. Higher T_1_ values observed in DHM1, DHM2, PHM3, DHSYM, and DHS candies reflected denser molecular packing and were consistent with their higher hardness values (Ozel et al., 2024).

Among all samples, *Chlorella*-enriched candies exhibited the lowest hardness, T_g_, and T_1_ values. These results reflected greater moisture uptake and matrix plasticization. This finding was consistent with previous reports describing a reduction in hardness following *Chlorella* incorporation in sugar-based systems (Shafiei and Mostaghim, 2022). Fracturability and brittleness results were consistent with TD-NMR and hardness data. This confirmed *Chlorella* candies as the most structurally fragile formulations.

The second-moment analysis revealed low proton mobility across all candy samples (Table 1). Crystallinity estimates showed that the control candy exhibited the highest crystalline fraction, whereas all algae-enriched candies exhibited similarly reduced crystallinity. These findings were further supported by XRD analysis, which confirmed the predominance of an amorphous structure with minimal crystalline domains (Kuzu et al., 2025).

### Antioxidant activity

Microalgal biomass contained diverse bioactive compounds that contributed to antioxidant capacity. In this study, *Dictyosphaerium* DHM1, DHM2, and *Pectinodesmus* PHM3 biomasses exhibited the highest radical scavenging activity (Table 3). However, the corresponding candies exhibited lower antioxidant values, which reflected partial degradation of heat-sensitive compounds during high-temperature processing. Similar reductions were reported previously for microalgae-enriched food systems (Batista et al., 2017).

**Table 3:** Antioxidant properties and bioactive compound content of microalgae biomass and their corresponding hard candies

	Samples	pFRAP mgTE/g	Radical Scavenging %	DPPH mgTE/g	Phenol mgGA/g	Flavonoid mgCHE/g
Microalgae powder	**Spk**	9.32 ± 0.02^A^	30.1 ± 0.28^F^	4.92 ± 0.07^B^	1.72 ± 0.04^B^	1.66 ± 0.04^E^
	**Ch**	8.49 ± 0.02^C^	47.2 ± 0.2^E^	3.96 ± 0.25^E^	1.33 ± 0.02^D^	1.86 ± 0.01^A^
	**Sp**	9.48 ± 0.01^B^	52.9 ± 0.6^D^	3.38 ± 0.1^F^	1.496 ± 0.01^C^	1.76 ± 0.01^B^
	**DHM1**	8.16 ± 0.04^D^	67.03 ± 0.21^C^	4.44 ± 0.03^D^	1.816 ± 0.09^B^	1.46 ± 0.02^C^
	**DHM2**	7.74 ± 0.02^E^	70.53 ± 0.15^A^	5.09 ± 0.02^A^	1.82 ± 0.07^B^	1.7 ± 0.03^B^
	**PHM3**	6.44 ± 0.04^F^	69.3 ± 0.2^B^	5.22 ± 0.05^A^	0.89 ± 0.08^B^	1.73 ± 0.04^B^
	**DHSYM**	6.1 ± 0.1^G^	65.63 ± 0.21^G^	5.4 ± 0.1^A^	1.98 ± 0.02^A^	1.53 ± 0.01^D^
	**DHS**	6.72 ± 0.04^H^	62.53 ± 0.15^H^	4.7 ± 0.02^C^	1.99 ± 0.02^A^	1.85 ± 0.03^A^
Microalgae candy	**C candy**	0.0^D^	0.0^I^	0.03 ± 0.05^D^	0.0^A^	0.017 ± 0.001^C^
	**Spk candy**	0.41 ± 0.02^B^	19.43 ± 0.21^F^	0.47 ± 0.06^AB^	0.025 ± 0.0^C^	0.02 ± 0.0^AB^
	**Ch candy**	0.38 ± 0.0^A^	14.43 ± 0.32^C^	0.4 ± 0.04^B^	0.045 ± 0.001^D^	0.02 ± 0.0^AB^
	**Sp candy**	0.41 ± 0.01^B^	16.87 ± 0.9^D^	0.532 ± 0.04^A^	0.013 ± 0.03^B^	0.02 ± 0.0^BC^
	**DHM1 candy**	0.37 ± 0.01^A^	13.66 ± 0.25^E^	0.21 ± 0.06^C^	0.086 ± 0.003^G^	0.02 ± 0.0^BC^
	**DHM2 candy**	0.38 ± 0.0^A^	13.62 ± 0.28^B^	0.21 ± 0.04^C^	0.062 ± 0.002^E^	0.02 ± 0.0^AB^
	**PHM3 candy**	0.43 ± 0.0^C^	15.21 ± 0.2^A^	0.23 ± 0.04^C^	0.08 ± 0.002^F^	0.02 ± 0.0^AB^
	**DHSYM candy**	0.37 ± 0.02^A^	13.56 ± 0.2^G^	0.193 ± 0.04^C^	0.08 ± 0.002^G^	0.02 ± 0.0^AB^
	**DHS candy**	0.37 ± 0.01^A^	14.27 ± 0.03^H^	0.27 ± 0.03^C^	0.09 ± 0.004^H^	0.02 ± 0.0^BC^

Analysis: pFRAP/Total reducing power (mg TE/g), Radical Scavenging activity (%), DPPH Radical scavenging (mg TE/g), Total phenolic content (mg GAE/g), Total flavonoid content (mg CE/g). Samples: *C* Control, *SPK* *Spirulina platensis* Kayseri, *Ch* *Chlorella vulgaris*, *Sp* *Spirulina platensis*, *DHM*1 *Dictyosphaerium* HM1, *DHM*2 *Dictyosphaerium* HM2, *PHM*3 *Pectinodesmus pectinus* HM3, *DHSYM* *Dictyosphaerium* HSYM, *DHS* *Dictyosphaerium* HS. Data represent mean ± SD (n = 3). Different uppercase letters within a column for each sample type (microalgae powder and microalgae candy) indicate significant differences between samples (p < 0.05, Tukey’s test)

^1^Values are presented as mean ± standard deviation (n = 3). Different subscript letters within the column indicate significant differences (p < 0.001; Tukey HSD test)

Antioxidant concentration measured by DPPH and pFRAP assay results were highest in *Chlorella* and *Spirulina* biomass, that were partially retained in the final candy formulations (Table 3). Antioxidant retention was influenced by initial concentration and algal composition. The higher lipid content of Pectinodesmus PHM3 provided partial protection to lipophilic antioxidants during thermal processing, whereas polar antioxidants exhibited greater susceptibility to degradation.

Although phenolic and flavonoid retention in candies was limited, algae-enriched formulations exhibited significantly higher radical scavenging activity than the control (Table 3). These results demonstrated enhanced antioxidant functionality derived from algal bioactive compounds. (Zhao et al., 2020).

### Estimated nutritional contents

Microalgal biomass exhibited notable compositional variation (Table 2). Protein content ranged from 32 to 51% (highest in *Chlorella*), carbohydrates were highest in *Dictyosphaerium* strains (31–34%), and lipids peaked in *Pectinodesmus* PHM3 (22%). The results were consistent with reported ranges for food-grade microalgae (Lupatini et al., 2017). In the corresponding candies (Table 1), carbohydrate content was slightly lower than in control candy (92–93% and 95% respectively). Protein content increased to 0.46–0.71% (highest with *Chlorella*), while lipids and other components increased to 0.39–3.79% (highest with PHM3), reflecting the composition of the source biomass. Moisture content remained between 2.98–5.27%, a range considered sufficient to support flavor stability, inhibit high crystallization, and preserve hardness in candies (Ozel et al., 2024). Mineral content was generally ≤ 0.95%, except in DHSYM (2.83%), suggesting higher mineral retention for the formulation. FTIR spectra depict –OH and –NH₂ functional groups (proteins/carbohydrates) supporting the analytical findings (Nandiyanto et al., 2019). Overall, microalgae supplemented sugar, protein, and lipid content. These results are consistent with established nutritional profiles of microalgae (Lupatini et al., 2017; Ursu et al., 2014).

### Preliminary cytocompatibility analysis

Preliminary cytocompatibility of algae-enriched hard candies was evaluated using MTT assay in HEK-293 cells. The viability of cells after exposure to algae candies provides an initial indication of compatibility under in-vitro conditions. The cell viability of all algae hard candy samples results indicated preliminary cytocompatibility. The results of cell viability for concentrations of hard candies 30%, 70%, and 100% are given in Table 1. The cell viability for algae candies was recorded at 81 to 86.7%. Similar results were observed in hard candies developed with herbal extracts assessed for cytotoxicity on W138 cell line (El-Sayed, 2023).

### Shelf life

The completed stability data was summarized in Table 4, which demonstrated that key physical properties color, moisture content, and hardness remain stable over a 3-month period. Antioxidant activity of algae hard candies declined gradually. Candies were stored in metallized polyester packaging under controlled conditions (18–22 °C, 45–60% RH, dark cabinet) to simulate optimal retail and storage environments.

**Table 4:** Shelf-life analysis of microalgae-enriched hard candies over a three-month storage period

Parameter	Months	C	Spk	Ch	Sp	DHM1	DHM2	PHM3	DHSYM	DHS	p-value
Hardness	Zero	15.5 ± 0.5^A^	15.67 ± 0.38^A^	14.33 ± 0.63^A^	15.25 ± 0.66^A^	15.75 ± 0.43^A^	15.42 ± 0.52^A^	15.75 ± 0.25^A^	15.75 ± 0.5^A^	15.92 ± 0.38^A^	0.123
	First	15.5 ± 0.5^AB^	15.67 ± 0.38^AB^	14.33 ± 0.63^B^	15.25 ± 0.66^AB^	15.75 ± 0.43^A^	15.41 ± 0.52^AB^	15.75 ± 0.25^A^	15.75 ± 0.5^A^	15.92 ± 0.38^A^	0.0307
	Second	15.5 ± 0.5^AB^	15.5 ± 0.5^AB^	14.25 ± 0.66^B^	15.1 ± 0.57^AB^	15.75 ± 0.43^A^	15.41 ± 0.46^AB^	15.74 ± 0.26^A^	15.73 ± 0.5^A^	15.86 ± 0.4^A^	0.0196
	Third	15.5 ± 0.5^AB^	15.42 ± 0.63^AB^	14.17 ± 0.52^B^	15 ± 0.66^AB^	15.75 ± 0.38^A^	15.4 ± 0.46^AB^	15.7 ± 0.26^A^	15.7 ± 0.5^A^	15.7 ± 0.37^A^	3.397
Moisture	Zero	3.6 ± 0.1^CD^	4.03 ± 0.2^BC^	5.27 ± 0.1^A^	4.3 ± 0.2^B^	2.98 ± 0.02^F^	3.05 ± 0.1^EF^	3.6 ± 0.1^D^	3.45 ± 0.3^DE^	4.06 ± 0.1^B^	< 0.001
	First	3.7 ± 0.01^CD^	4.03 ± 0.2^BC^	5.27 ± 0.1^A^	4.3 ± 0.2^B^	2.98 ± 0.02^F^	3.05 ± 0.1^EF^	3.6 ± 0.1^D^	3.45 ± 0.3^DE^	4.06 ± 0.1^B^	< 0.001
	Second	3.64 ± 0.1^C^	4.03 ± 0.2^B^	5.28 ± 0.0^A^	4.3 ± 0.1^B^	2.98 ± 0.1^E^	3.05 ± 0.0^DE^	3.6 ± 0.1^C^	3.45 ± 0.3^CD^	4.06 ± 0.0^B^	< 0.001
	Third	3.64 ± 0.03^C^	4.03 ± 0.1^B^	5.28 ± 0.1^A^	4.3 ± 0.1^B^	2.98 ± 0.1^E^	3.05 ± 0.0^DE^	3.6 ± 0.1^C^	3.45 ± 0.3^CD^	4.06 ± 0.0^B^	< 0.001
Delta E	Zero	0.21 ± 0.03^F^	0.14 ± 0.05^G^	0.3 ± 0.01^E^	0.3 ± 0.0^E^	0.2 ± 0.0^F^	1.0 ± 0.01^A^	0.54 ± 0.0^D^	0.58 ± 0.0^B^	0.81 ± 0.01^C^	< 0.001
	First	0.95 ± 0.42^B^	0.2 ± 0.05^F^	0.3 ± 0.0^E^	0.3 ± 0.0^E^	0.1 ± 0.01^G^	1.0 ± 0.01^A^	0.41 ± 0.0^D^	0.67 ± 0.0^C^	0.9 ± 0.01^B^	< 0.001
	Second	0.5 ± 0.12^B^	0.32 ± 0.05^DE^	0.1 ± 0.0^F^	0.3 ± 0.01^E^	0.0 ± 0.01^G^	0.4 ± 0.08^CD^	0.3 ± 0.03^E^	0.6 ± 0.05^A^	0.4 ± 0.01^C^	< 0.001
	Third	0.4 ± 0.1^F^	0.61 ± 0.05^D^	0.05 ± 0.01^H^	0.4 ± 0.02^F^	0.2 ± 0.02^G^	0.5 ± 0.01^E^	1.46 ± 0.2^A^	1.38 ± 0.1^B^	0.91 ± 0.03^C^	< 0.001
Antioxidant	Zero	0.04 ± 0.0^E^	0.48 ± 0.04^AB^	0.39 ± 0.04^BC^	0.55 ± 0.06^A^	0.21 ± 0.07^D^	0.21 ± 0.04^D^	0.23 ± 0.04^D^	0.22 ± 0.04^D^	0.28 ± 0.04^CD^	< 0.001
	First	0.03 ± 0.0^E^	0.47 ± 0.06^AB^	0.38 ± 0.04^BC^	0.53 ± 0.07^A^	0.19 ± 0.08^D^	0.2 ± 003^D^	0.23 ± 0.03^D^	0.22 ± 0.02^CD^	0.28 ± 0.01^CD^	< 0.001
	Second	0.03 ± 0.0^E^	0.47 ± 0.01^A^	0.35 ± 0.03^B^	0.48 ± 0.01^A^	0.18 ± 0.01^D^	0.19 ± 0.0^D^	0.22 ± 0.01^CD^	0.21 ± 0.0^CD^	0.26 ± 0.01^C^	< 0.001
	Third	0.02 ± 0.0^D^	0.42 ± 0.0^A^	0.34 ± 0.03^B^	0.47 ± 0.01^A^	0.15 ± 0.0^C^	0.18 ± 0.01^C^	0.21 ± 0.01^C^	0.21 ± 0.01^C^	0.21 ± 0.0^C^	< 0.001

Parameters measured include: Hardness (kgF), Moisture content (%), Color change (ΔE), and Antioxidant capacity (mg Trolox Equivalents [TE]/g). Data represents standard deviation (SD), n = 3. For each parameter and storage time, different uppercase letters indicate statistically significant differences between the candy samples (p < 0.05). Statistical analysis was performed using one-way ANOVA followed by Tukey’s HSD post-hoc test. Samples are coded as: *C* Control, *Spk* *Spirulina platensis* Kayseri, *Ch* *Chlorella vulgaris*, *Sp* *Spirulina platensis*, *DHM*1, *Dictyosphaerium* HM1, *DHM*2 *Dictyosphaerium* HM2, *PHM*3 *Pectinodesmus pectinus* HM3, *DHSYM* *Dictyosphaerium* HSYM, *DHS* *Dictyosphaerium* HS

^1^Values are presented as mean ± standard deviation (n = 3). Different superscript letters within the column indicate significant differences (Tukey HSD test)

^2^Significance was defined as p < 0.05 (significant), p < 0.01 (highly significant), and p < 0.001 (extremely significant)

Hardness (measured in kgF) exhibited a small and not statistically significant decrease in the *Spirulina* and *Chlorella* enriched candies, while the control maintained its textural integrity. Color parameters and moisture remained effectively unchanged throughout the storage duration. In contrast, antioxidant capacity, as measured by the DPPH assay (mg TE/g), demonstrated a gradual decline over time. This indicates that while packaging and storage preserved structural and visual attributes. Phenolic or antioxidant compounds are more vulnerable to slow degradation. This observation aligns with findings from similar confectionery systems such as white tea–based candies. The white tea-based candies lost bioactive quality when stored at 4 and 22 °C for four months (Šeremet et al., 2020). Antioxidant activity, measured via DPPH (mg TE/g), declined gradually over time. A trend commonly observed in various food systems, including pumpkin candies and other plant-based confections (Muzzaffar et al., 2016). The decline however was 10 to 25% loss of total antioxidant capacity in three months.

Hard candies are known for their glassy, low-moisture structure, which limits molecular mobility and helps preserve mechanical as well as visual quality over time, provided moisture ingress is effectively prevented (Ozel et al., 2024). However, antioxidant compounds, compared to structural components and colorants, are more chemically labile and prone to oxidation or polymerization during storage, even under ambient thermal conditions.

The current study indicates significant changes in physical qualities and biochemical profile by the addition of microalgae biomass to hard candies. The physicochemical results indicate the incorporation of algae maintained structural integrity of the candies. A comparison with control candies confirms algae incorporation not compromising texture. The nutritional composition of hard candies revealed higher protein, mineral, and lipid content in microalgae-supplemented hard candies as compared with control candies. XRD and TD-NMR analysis proved low crystallinity of hard candies. The structure and texture analysis revealed the addition of algae not significantly increasing or decreasing candy hardness apart from *Chlorella*. The addition of microalgae elevates total antioxidant content of hard candies. Presence of antioxidants in the candies indicate partial resistance of algal bioactive compounds to high-temperatures processing. Organoleptic analysis indicated higher visual and textural acceptance for *Chlorella* and *Spirulina* candies. The hardness, moisture, and color of candy do not change between 3 months of storage, but antioxidant content is reduced which may be attributed to moisture present within the packaging. Despite stable moisture, hardness, and color values over the storage period, a gradual decline in antioxidant activity was observed. This discrepancy reflects the differing stability mechanisms of physical versus chemical quality parameters. The high-sugar, low-moisture matrix of hard candies effectively preserves texture and color, whereas bioactive compounds from algae remain susceptible to oxidative and structural degradation over time, even in physically stable products.

In conclusion, this study demonstrates the feasibility of incorporating diverse microalgae species into hard candies, providing new knowledge on how species-specific algal composition dictates functional properties. *Chlorella* and *Spirulina* powders had favorable organoleptic scores (based on visual/texture evaluation) and antioxidant retention. The study evaluated structural attributes such as molecular mobility, crystallinity and texture of candies by TD-NMR, XRD, DSC, and SEM. The 3-month shelf life of candies was evaluated for understanding the structural and chemical degradation. The candies maintained structural integrity and key physical properties over a 3-month shelf life, although antioxidant activity gradually declined. Preliminary cytocompatibility was indicated using a HEK-293 cell model, though safety assessment in additional cell lines is warranted. Importantly, claims regarding taste and full consumer acceptability are limited as the formal sensory taste trials needs to be conducted, which represent an essential next step for product development. This work serves as a foundational reference for the rational design of algae-fortified functional confectionery. Moreover, further evaluation through human sensory trials, consumer acceptance, and other properties of these hard candies is a necessary future step for successful algal food production. Algae hard candies in this study can be considered as functional foods.

## Supplementary Information

Below is the link to the electronic supplementary material.

Supplementary file1 (DOCX 81 KB)

## Data Availability

The datasets generated and analysed during the current study are available from the corresponding author upon reasonable request.

## Abbreviations

BSA: Bovine serum albumin
HC: Hard candies
NMR: Nuclear magnetic resonance
RBC: Red blood cells
XRD: X-ray diffraction
