# Supplementary material for: Evaluating the impact of microalgae powder on physicochemical and functional properties of hard candies
Source: Food Sci Biotechnol. 2026 Jun 3;35(8):2191–205. doi: 10.1007/s10068-026-02174-0 (PMC13283091; doi:10.1007/s10068-026-02174-0)
Supplement: Supplementary file 1 — Supplementary file1 (DOCX 81 KB) [file 10068_2026_2174_MOESM1_ESM.docx]

**Evaluating the Impact of Microalgae Powder on Physicochemical and Functional Properties of Hard Candies**

**Short Title:** Characteristics of Algal-Derived Hard Candies

Shafia Maryam^a^ , Berkay Berk^b,e^, Hilmi Eriklioglu^b^, Baris Ege Gulenc^b^, Kosain Kousar^a^, Mecit Halil Oztop^b^, Muhammad Qasim Hayat^c^, Sarper Dogdu^d^, Mehmet Ali Marangoz^d^ and Hussnain Ahmed Janjua^a^

^a^Department of Microbiology and Biotechnology, Atta-ur-Rahman School of Applied Biosciences, National University of Science and Technology, Islamabad, Pakistan. 44000

^b^Food Engineering Department, Middle East Technical University, Ankara, Turkey. 06800

^c^Department of Plant Biotechnology, Atta-ur-Rahman School of Applied Biosciences, National University of Sciences and Technology, Islamabad, Pakistan. 44000

^d^Durukan Sekerleme, Ankara, Türkiye. 06935

^e^Izmir Institute of Technology, Department of Food Engineering, Izmir/Türkiye. 35433

Corresponding Authors: Dr. Hussnain Ahmed Janjua [janjua.hussnain@gmail.com](mailto:janjua.hussnain@gmail.com); [hussnain.janjua@asab.nust.edu.pk](mailto:hussnain.janjua@asab.nust.edu.pk); Dr. Mecit Halil Oztop [mecit@metu.edu.tr](mailto:mecit@metu.edu.tr)

**Tables**

**Table S1.** One-way ANOVA results comparing physicochemical, TD-NMR, antioxidant, compositional and cytotoxicity assay results of algae biomass and hard candies. F- and p-values are shown for each parameter, with significance levels denoted as: ***p < 0.001 (extremely significant), **p < 0.01 (highly significant), *p < 0.05 (significant), and NS (not significant, p ≥ 0.05). Tukey’s post-hoc tests confirmed significant differences between sample types for all parameters except crystallization percentage (p = 0.0658). Color ΔE and fracturability showed the highest F-values (F > 200), indicating extreme sensitivity to treatment effects.

| **Parameter** | **F-Value** | **p-Value** | **Significance** | **Tukey's Result** |
| --- | --- | --- | --- | --- |
| **Color L (candy)** | 250173 | < 0.0001 | *** | Extremely significant |
| **Color a (candy)** | 20201 | < 0.0001 | *** | Extremely significant |
| **Color b (candy)** | 129709 | < 0.0001 | *** | Extremely significant |
| **Color L (algae)** | 111.42 | < 0.0001 | *** | Extremely significant |
| **Color a (algae)** | 297.11 | < 0.0001 | *** | Extremely significant |
| **Color b (algae)** | 27465 | < 0.0001 | *** | Extremely significant |
| **Particle size** | 62.09 | < 0.0001 | *** | Extremely significant |
| **Hardness** | 2.857 | 0.0307 | * | Significant |
| **Brittleness** | 187.3 | < 0.0001 | *** | Extremely significant |
| **Fracturability** | 227.1 | < 0.0001 | *** | Extremely significant |
| **Glass Transition Temp** | 14.76 | < 0.0001 | *** | Extremely significant |
| **Total Soluble Solids** | 4.911 | 0.0024 | ** | Highly significant |
| **T1 (Texture)** | 79.9 | < 0.0001 | *** | Extremely significant |
| **Second Moment** | 3.848 | 0.0084 | ** | Significant |
| **Crystallization %** | 2.320 | 0.0658 | NS | Not significant |
| **pFRAP (Algae)** | 16452 | < 0.0001 | *** | Extremely significant |
| **Radical Scavenging**  **% (Algae)** | 23622 | < 0.0001 | *** | Extremely significant |
| **DPPH (Algae)** | 42.89 | < 0.0001 | *** | Extremely significant |
| **Phenolics (Algae)** | 431.5 | < 0.0001 | *** | Extremely significant |
| **Flavonoids (Algae)** | 1,588 | < 0.0001 | *** | Extremely significant |
| **pFRAP (Candies)** | 589.5 | < 0.0001 | *** | Extremely significant |
| **Radical Scavenging**  **% (Hard candies)** | 682.6 | < 0.0001 | *** | Extremely significant |
| **DPPH (Candies)** | 22.98 | < 0.0001 | *** | Extremely significant |
| **Phenol (Candies)** | 541.9 | < 0.0001 | *** | Extremely significant |
| **Flavonoid (Candies)** | 1.733 | < 0.0001 | *** | Extremely significant |
| **Carbohydrates (Algae)** | 5205 | < 0.0001 | *** | Extremely significant |
| **Proteins (Algae)** | 10760 | < 0.0001 | *** | Extremely significant |
| **Lipids (Algae)** | 2555 | < 0.0001 | *** | Extremely significant |
| **Minerals (Algae)** | 377.6 | < 0.0001 | *** | Extremely significant |
| **Other Components**  **(Algae)** | 378.7 | < 0.0001 | *** | Extremely significant |
| **Carbohydrates**  **(Candies)** | 29.02 | < 0.0001 | *** | Extremely significant |
| **Proteins (Candies)** | 1004 | < 0.0001 | *** | Extremely significant |
| **Minerals (Candies)** | 6,400 | < 0.0001 | *** | Extremely significant |
| **Moisture (Candies)** | 309.7 | < 0.0001 | *** | Extremely significant |
| **MTT Assay (100%)** | 42.69 | < 0.0001 | *** | Extremely significant |
| **MTT Assay (70%)** | 18.79 | < 0.0001 | *** | Extremely significant |
| **MTT Assay (30%)** | 3.278 | 0.0173 | * | Significant |

**Table S2.** One-way ANOVA results comparing changes in quality parameters of hard candies during 3-month storage. F- and p-values are shown for each parameter, with significance levels denoted as: ***p < 0.001 (extremely significant), **p < 0.01 (highly significant), *p < 0.05 (significant), and NS (not significant, p ≥ 0.05).

| **Parameter** | **0 Months (F/p)** | **1 Month (F/p)** | **2 Months (F/p)** | **3 Months (F/p)** |
| --- | --- | --- | --- | --- |
| **Hardness** | F=1.899 (p=0.123) | F=2.857 (p=0.0307)* | F=3.185 (p=0.0196)* | F=3.397 (p=0.0148)* |
| **Moisture** | F=67.17 (p<0.0001)*** | F=68.01 (p<0.0001)*** | F=79.75 (p<0.0001)*** | F=92.68 (p<0.0001)*** |
| **Color ΔE** | F=2676 (p<0.0001)*** | F=2188 (p<0.0001)*** | F=307 (p<0.0001)*** | F=2828 (p<0.0001)*** |
| **Antioxidants** | F=38.06 (p<0.0001)*** | F=34.48 (p<0.0001)*** | F=134.2 (p<0.0001)*** | F=111.4 (p<0.0001)*** |

**Figure**

| A. |
| --- |
| 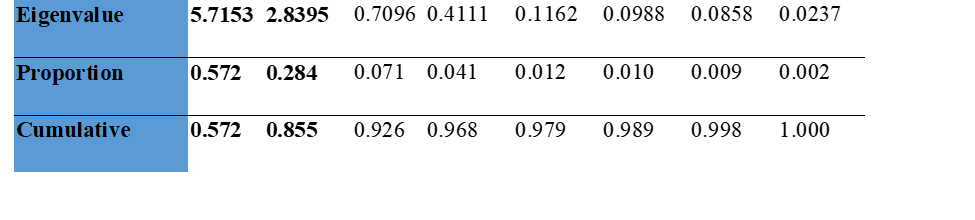 B. |

**Fig. S1** Organoleptic analysis results. **(A)** Organoleptic Results of one control and eight algae hard candies. The nine parameters of aroma, shine, color, hue of green, transparency, texture, hardness, stickiness, acceptability and expectations. **(B)** Eigen analysis of the correlation matrix. Samples: **Control**, Control; **Spirullina Kayseri**, *Spirulina platensis* Kayseri; **Chlorella**, *Chlorella vulgaris*; **Spirullina**, *Spirulina Platensis*; **DHM1**, *Dictyosphaerium* HM1; **DHM2**, *Dictyosphaerium* HM2; **PHM3**, *Pectinodesmus pectinus*; **DHSYM** *Dictyosphaerium* HSYM; **DHS**, *Dictyosphaerium* HS.
